# In-hospital mortality of patients with severe left ventricular dysfunction undergoing coronary artery bypass grafting in Iranian population

**DOI:** 10.1186/s13019-022-01906-7

**Published:** 2022-06-20

**Authors:** Ahmadali Khalili, Mehran Rahimi, Naser Khezerlouy-aghadam, Fariborz Akbarzadeh, Mohammadreza Taban-sadeghi

**Affiliations:** Cardiovascular Research Center, Tabriz University of Medical Sciences, Daneshgah Street, Tabriz, Eastern Azerbaijan Iran

**Keywords:** CABG, Heart failure, Mortality and morbidity, In-hospital complications

## Abstract

**Background:**

Historically, coronary artery bypass grafting is associated with a higher mortality rate in patients with severe heart failure. This study aimed to assess the in-hospital mortality of CABG in patients with severe heart failure in Iranian patients and to identify factors associated with adverse outcomes.

**Methods:**

This retrospective descriptive study enrolled patients with severe heart failure who underwent coronary artery bypass surgery from 2015 to 2020 in Madani Hospital, affiliated with Tabriz University of Medical Sciences.

**Results:**

A total of 865 consecutive patients with a mean age of 60.65 ± 10.00 were enrolled in the study. Of all participants, 175 were female (20.4%), and 684 were male. The overall mortality rate was 9.5%. In the univariate analysis, predictors of ICU mortality were age, female sex, DM, and renal failure (*P* value < 0.05). None of the factors studied was an independent predictor of ICU mortality in the multivariate analysis.

**Conclusion:**

This study established that although coronary artery bypass surgery is reported to have low mortality and postoperative morbidity in patients with severe heart failure, there are still centers that face higher mortality rates in these patients. Improving these patients' outcomes would be possible through identifying the associated risk factors and pre-and postoperative management.

## Background

Aggressive medical treatment in patients with end-stage ischemic cardiomyopathy and severe heart failure has unsatisfactory outcomes in terms of long-term survival [1]. Heart transplant, surgical revascularization, or device implantation are the remaining options for the patients. Heart transplants and devices are limited by the small number of donors and the high cost of devices, respectively [2].

Coronary artery bypass grafting (CABG) aids people with coronary artery disease to live longer, and enhances their quality of life, and in patients with severe heart failure, CABG has been shown to decrease the mortality rate [3]. However, high morbidity and mortality rates make surgery a significant challenge among these patients. Severe heart failure, defined as left ventricular ejection fraction (LVEF) below 35%, was associated with a considerable postoperative mortality rate [4]. Most patients with severe heart failure have a variety of risk factors such as diabetes, hypertension, and hyperlipidemia that increase the mortality rate in these patients [5]. But, there has been a substantial decrease in the mortality rate in recent years due to advancements in surgical techniques and postoperative care [6].

This study aimed to assess in-hospital mortality and morbidity in patients with severe heart failure in Iranian patients undergoing CABG and identify factors associated with adverse outcomes.

## Methods

This retrospective descriptive study enrolled patients with severe heart failure who underwent coronary artery bypass surgery from 2015 to 2020 in Madani Hospital, affiliated with Tabriz University of Medical Sciences.

Inclusion criteria consisted of patients with severe heart failure described as LVEF below 35%, treated with coronary artery bypass surgery. Exclusion criteria were simultaneous cardiac valve replacement surgery, history of cardiac pacemaker implantation, and patients’ unwillingness to participate in the study.

Heart failure was initially diagnosed via echocardiography by a cardiology specialist. Patients had undergone surgery performed by one of 10 surgeons of our center. Data regarding patients’ age, sex, weight, height, familial history, medical history, including diabetes mellitus (DM), hypertension (HTN), hyperlipidemia (HLP), renal failure (RF), history of opioid use, used cigarette counts per day, serum creatinine (Cr) concentrations, and total days of hospitalization in an intensive care unit (ICU) and general ward were obtained through medical recordings.

Data were collected from pre-operative evaluations, such as electrocardiography (including heart rate (HR), corrected QT interval (QTc), presence of bundle branch block, location of myocardial infarction (MI)), echocardiography (including the presence of left ventricular ejection fraction (EF), end-systolic and end-diastolic left ventricular internal diameter, and left ventricular regional wall motion abnormalities such as akinesia or hypokinesia), and angiography (including the site of lesion or stent in the left main coronary (LMC), left anterior descending (LAD), left circumflex (LCX), and right coronary artery (RCA)).

Postoperative data regarding mortality rate in ICU and ward, complications such as hemorrhage, cardiac arrest, and mediastinitis, intracardiac device (ICD), and pacemaker implantation requirement, and outcome at discharge were collected.

### Statistical analysis

The data were analyzed using SPSS software version 22.0 (SPSS INC., IBM Corporation, Chicago, IL). The Kolmogorov–Smirnov test was used to evaluate normality. Qualitative data were reported as frequency (percentage). Quantitative data were presented as mean ± standard deviation (SD). A chi-squared test was used to examine the relationship between qualitative data, and a Student t-test was used to investigate the relationship between quantitative data. A *P* value less than 0.05 was considered to establish a statistically significant association. Multivariate regression analyses were used to estimate the relationship between risk factors and patients’ mortality.

## Results

A total of 865 consecutive patients were enrolled in the study. Of all participants, 175 were female (20.4%), and 684 were male. The most common comorbidity in the patients was hypertension (56.9%). Akinesia was more common than hypokinesia (45.9% vs. 41.2%). Table 1 shows the patients’ demographic characteristics, angiography results, trans-thoracic echocardiography, and electrocardiography.

**Table 1:** Demographic characteristics of the patients (N = 865)

Characteristics	Number (percent%) or mean ± standard deviation
Age (year)	60.65 ± 10.00
Male sex	684 (79.1%)
DM	356 (41.2%)
HTN	492 (56.9%)
Renal failure	260 (30.1%)
Smoker	352 (40.7%)
HLP	130 (15.0%)
Height (cm)	165.33 ± 9.99
Weight (kg)	74.36 ± 13.02
Familial history	34 (3.9%)
Serum creatinine concentration (mg/dl)	1.40 ± 4.56
Duration of ICU hospitalization (day)	6.27 ± 6.63
Duration of ward hospitalization (day)	4.60 ± 2.90
*ECG findings*	
BBB	124 (14.3%)
Anterior MI	409 (47.3%)
Inferior MI	314 (36.4%)
Lateral MI	239 (27.6%)
Posterior MI	64 (7.4%)
Rate	84.93 ± 16.13
QTC	414.85 ± 61.66
*Echocardiography findings*	
Akinesia	397 (45.9%)
Hypokinesia	356 (41.2%)
LVEF	28.89 ± 8.02
LV size (systolic, cm)	6.31 ± 7.70
LV size (diastolic, cm)	3.77 ± 1.61
*Angiography findings*	
LMC	131 (15.1%)
LMC stent	9 (1.0%)
LAD	839 (97.0%)
LAD stent	17 (2.0%)
LCX	654 (75.6%)
LCX stent	21 (2.4%)
RCA	720 (83.2%)
RCA stent	18 (2.1%)

*DM* diabetes mellitus, *HTN* hypertension, *HLP* hyperlipidemia, *BBB* bundle branch block, *MI* myocardial infarction, *QTC* corrected QT interval, *LVEF* left ventricle ejection fraction, *LMC* left main coronary artery, *LAD* left anterior descending artery, *LCX* left circumflex artery, *RCA* right coronary artery

The overall mortality rate was 9.5%, and 97.6% of the mortality occurred in the ICU. The mortality rate was significantly lower in the off-pump surgery group (5.92% vs. 12.9%, *P* value = 0.005). We observed ICU hemorrhage in 17.8% of the subjects (the most common complication). Patients’ outcomes following surgery are presented in Table 2.

**Table 2:** Post procedure findings of the patients

Post procedure findings	
ICU hemorrhage	154 (17.8%)
Mediastinitis	3 (0.3%)
Pace maker	1 (0.1%)
ICD	11 (1.3%)
On-pump CABG	443 (51.2%)
Cardiac arrest in ICU	44 (5.1%)
ICU mortality	80 (9.2%)
Ward mortality	2 (0.2%)
Cardiac arrest in ward	34 (3.9%)
Overall hospital mortality	82 (9.5%)

*CABG* coronary artery bypass grafting, *ICU* intensive care unit, *ICD* intracardiac device

Table 3 shows the regression analysis results of risk factors based on ICU mortality. While in the univariate analysis, predictors of ICU mortality were age, female sex, DM, and renal failure, none of the factors studied was an independent predictor of ICU mortality in the multivariate analysis.

**Table 3:** Univariate and Multivariate analysis of factors associated with ICU mortality

Factor	Univariate				Multivariate			
	HR	95% CI for HR		*p* value	HR	95% CI for HR		*p* value
		Lower	Upper			Lower	Upper	
Age	1.036	1.012	1.062	0.004*	1.035	1.007	0.012	1.062
Sex	2.222	1.361	3.629	0.001*	2.173	1.107	0.024	4.266
DM	1.790	1.135	2.821	0.012*	1.621	0.994	0.053	2.646
HTN	1.041	0.658	1.646	0.864	.690	0.416	0.149	1.142
Familial History	0.906	0.271	3.030	0.873	.990	0.284	0.988	3.448
Height	0.992	0.970	1.013	0.441	1.018	0.985	0.285	1.052
Weight	0.985	0.968	1.003	0.106	0.990	0.970	0.370	1.012
Renal failure	2.043	1.287	3.242	0.002*	1.973	1.215	0.006	3.204

*HR* hazard ratio, *CI* confidence interval, *DM* diabetes mellitus, *HTN* hypertension

*P value < 0.05

## Discussion

Our study draws a sample of patients with severe heart failure undergoing CABG. We demonstrated that the mortality rate in our setting is still high. Mediastinitis, pacemaker implantation requirement, cardiac arrest in ICU, and cardiac arrest in the ward were present in less than 10% of the patients. Age, female sex, DM, and renal failure were predictors of ICU mortality in patients. Although the mortality in this study is much higher than the mortality in the developed and even developing countries, our results depict a large sample of patients in a developing country that suffers from financial problems (Iran, in recent years, has faced difficult times because of sanctions and mismanagements).

CABG is commonly known to have higher operative mortality and reduced survival in patients with LVEF of less than 35% compared with those with normal ventricular function. Studies have reported a mortality rate of 5% to 20% in patients with heart failure undergoing CABG [7–10]. However, most studies performed in more recent years (after 2000) reported decreased mortality rate of less than 5% [11–14]. This improvement in the operative outcome of patients with poor left ventricular function is multifactorial. The survival of these high-risk patients has been benefited from advances in peri- and postoperative managements of comorbid risk factors and myocardial protection strategies such as vasodilator therapy, cardioplegic infusion, use of the postoperative intra-aortic balloon, improved techniques of anesthetic induction, epi-aortic scanning, and intensive insulin therapy [15, 16]. Despite these improvements, our study showed a mortality rate of 9.5%.

The choice of off-pomp CABG in patients with two or more risk factors was corroborated by various studies that documented the benefits of off-pomp CABG over on-pomp CABG in patients with risk factors and comorbidities such as ascending aorta atherosclerotic disease, renal failure and peripheral vascular disease, and elderly patients [17, 18]. Overall, the off-pomp CABG procedure was performed in 48.2% of patients.

In a study on the interactive effects of age on survival after coronary artery surgery, Ramanathan et al. concluded that younger patients have decreased survival rates. The hypothesis was that the same factors resulting in significant atherosclerosis at younger ages might reduce the survival rate of these patients [19]. Other studies have also explored the effect of age on the mortality rate and survival of patients undergoing CABG. The general conclusion was an increased mortality rate with advancing age [20, 21]. The mean age of patients in our study was 60.65 ± 10.00 years, and as expected, age was a predictor of a higher mortality rate in patients with severe heart failure undergoing surgery.

There are conflicting results regarding the effect of DM on survival after coronary artery surgery [22–24]. These conflicts might be due to various factors such as adequacy of DM control, the extent of coronary artery disease, and surgical techniques [25, 26]. Furthermore, patients with DM are more likely to have comorbid diseases such as chronic renal failure, peripheral vascular disease, and more extensive coronary artery disease than the nondiabetic group. The diabetic population represented 41.2% of the total participants in our study. We concluded that DM is associated with higher ICU mortality rate in patients with severe heart failure.

Additionally, female sex and renal failure have been identified as independent predictors of mortality [14, 27, 28]. Our results were in line with previous studies, reporting that age and sex are associated with ICU mortality in patients with LVEF less than 35%.

### Limitations

This is a descriptive study, and results are limited in their application. The limitations of the present study included the absence of control group and a short follow-up duration. Moreover, this study did not evaluate cost-analysis, postoperative EF, quality of life, late complications, or causes of death.

## Conclusion

This study established that although coronary artery bypass surgery is reported to have low mortality and postoperative morbidity in patients with severe heart failure, there are still centers that face higher mortality rates in these patients. Improving these patients' outcomes would be possible through identifying the associated risk factors and pre-and postoperative management.

## Data Availability

The datasets used and analyzed during the current study are available from the corresponding author on reasonable request.

## Abbreviations

CABG: Coronary artery bypass grafting
DM: Diabetes mellitus
HLP: Hyperlipidemia
HTN: Hypertension
ICU: Intensive care unit
LVEF: Left ventricular ejection fraction
